# Does practice of meniscus surgery change over time? A report of the 2021 ‘THE MENISCUS’ Webinar

**DOI:** 10.1186/s40634-021-00365-8

**Published:** 2021-06-26

**Authors:** Christophe Jacquet, Caroline Mouton, Roland Becker, Hideyuki Koga, Matthieu Ollivier, Peter Verdonk, Philippe Beaufils, Romain Seil

**Affiliations:** 1 Department of Orthopedic Surgery and Traumatology St. Marguerite Hospital, Institute for Movement and Locomotion (IML), Marseille, France; 2 Sports Clinic, Centre Hospitalier de Luxembourg-Clinique d’Eich, Luxembourg, Luxembourg; 3 Luxembourg Institute of Research in Orthopaedics, Sports Medicine and Science, Luxembourg, Luxembourg; 4 Department of Orthopaedics and Traumatology, Brandenburg University Theodor Fontane, Hochstrasse 26, 14776 Brandenburg, Germany; 5 Department of Joint Surgery and Sports Medicine, Graduate School of Medical and Dental Sciences, Tokyo Medical and Dental University, Tokyo, Japan; 6 ORTHOCA, Antwerp, Belgium; 7 Department of Orthopaedic Surgery, Centre Hospitalier de Versailles André Mignot, 177 rue de Versailles, 78150 Le Chesnay, France; 8 Human Motion, Orthopaedics, Sports Medicine and Digital Methods, Luxembourg Institute of Health, Luxembourg, Luxembourg

**Keywords:** MENISCUS Webinar, Meniscus Repair, Meniscectomy, ESSKA consensus

## Abstract

**Purpose:**

The aim of this paper was to report the results presented in the session “Does practice of meniscus surgery change over time?” of the 2021 MENISCUS webinar held online on January 30^th^ 2021.

**Method:**

During the 2021 MENISCUS webinar, an evaluation of meniscus surgery practices was performed by analyzing: (1) The presentation of the results of a survey conducted among ESSKA members and assessing their current practices in the field of meniscus surgery, (2) Four reports by national experts analyzing the trends in Arthroscopic Partial Meniscectomy (APM) and meniscus repair procedures in their respective countries (France, Belgium, Germany and Japan).

**Results:**

(1) ESSKA Survey: Among the 461 respondents, 75% of surgeons claimed to perform more meniscus repairs and 85% less APM than 5 years ago. In ACL-associated meniscus injuries, a majority of surgeons (60%) indicated to perform a meniscal resection in less than 25% of cases. 25% declared to perform meniscus repair in ACL-associated meniscus injuries in less than 25% of cases and 37% in more than 50% of cases. Half of the respondents repair medial or lateral root tears in less than 25% of cases. Less than 20% of respondents were not familiar with the ESSKA consensus.

(2) National trends: In France, between 2005 and 2017, the APM rate decreased by 21.4%, while the repair rate increased by 320%. In Belgium, between 2007 and 2017, the APM rate decreased by 28.6%. In Germany, between 2010 and 2017 the number of APM decreased by 30%, while the number of repair procedures increased by 55%. Finally, in Japan, between 2011 and 2016, the APM ratio (APM/meniscus procedures) decreased by 16% from 91 to 75% while the repair ratio increased from 9 to 25%.

**Conclusion:**

The 2021 ESSKA members' survey as well as statistics from 4 specifically examined countries (Belgium, France, Germany and Japan) suggest there has been a significant shift over the last years in the surgical management of meniscal lesions towards less APM and more conservative treatments.

## Introduction

A better understanding of the role of meniscus anatomy, its pathogenesis and the downsides of meniscectomy has led to the development of the “meniscus preservation” concept over the last few decades. In 2016, ESSKA initiated the European Meniscus Consensus Project [3]. It provided a reference frame for the management of Degenerative Meniscus Lesions (DML’s), based both on scientific literature and balanced expert opinion. The proposed decisional algorithm introduced Arthroscopic Partial Meniscectomy (APM) not as a first, but as a second line treatment of DML’s in symptomatic patients. In 2018, a new ESSKA meniscus consensus [7] was published to present recommendations for the treatment of acute traumatic meniscus tears (ATMT`s). The conclusion of this second consensus was that preservation of the meniscus should be the first line treatment whenever possible, due to the fact that clinical and radiological long-term outcomes are worse after APM than after meniscus preservation [8, 13, 15]. The consensus clearly stated that numerous traumatic meniscus injuries which were previously considered to be irreparable should be repaired.

The aim of this paper was to report the results presented in the session “Does practice of meniscus surgery change over time?” of the 2021 MENISCUS webinar held online on January 30^th^ 2021. The authors’ hypothesis was that, according to ESSKA members report of their practice and to national trends, the amount of meniscal repair procedures has increased in recent years, whereas the trend for APM has decreased.

## Method

During the 2021 MENISCUS webinar, in the section: “Does practice of meniscus surgery change over time?”, an evaluation of meniscus surgery practices was performed by analyzing:

- 1) The results of a survey conducted among ESSKA members. In order to assess the impact of the Consensus Projects on practice among ESSKA members, ESSKA performed a survey in conjunction with the Organizing Committee of “The Meniscus Congress”. This online survey included 11 questions. It was sent to ESSKA members and affiliated societies on December 3^rd^ 2020 and responses were collected until January 21^st^ 2021.
- 2) Four reports by national experts analyzing the trends in APM and meniscus repair procedures in their respective countries (France, Belgium, Germany and Japan).

## Results

### ESSKA survey

**Fig. 1:**
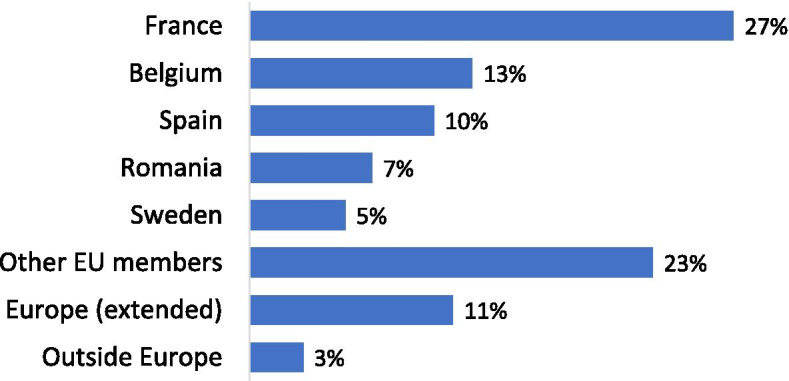
Nationality of ESSKA survey respondents

Four hundred and sixty-one ESSKA members from around the world participated in the survey, 86% came from countries of the European union (Fig. 1). The work setting of respondents was homogeneously distributed: 61% of respondents worked in an academic (35%) or a non-academic (26%) hospital and 38% of them worked in a private clinic.

**Fig. 2:**
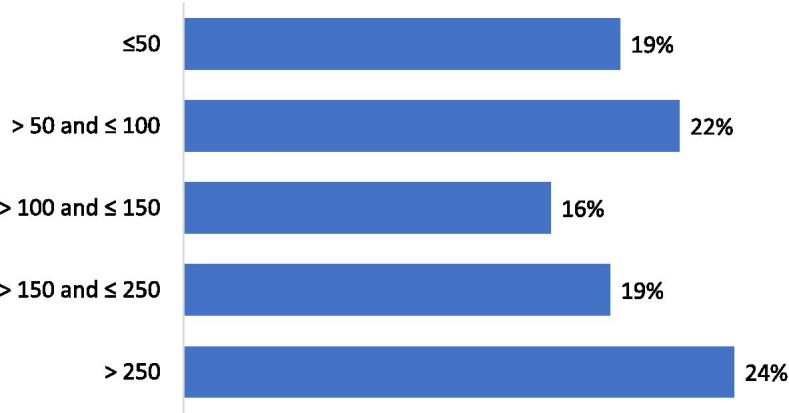
Answers to the question: “ How many knee arthroscopies do you perform per year?”

**Fig. 3:**
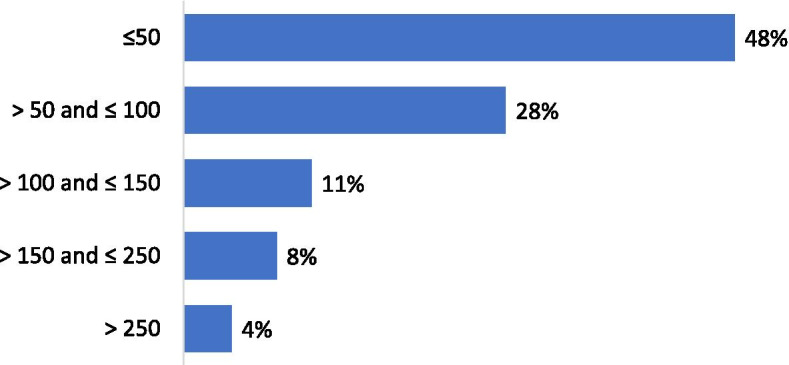
Answers to the question: “How many isolated meniscal procedures do you perform per year?”

**Fig. 4:**
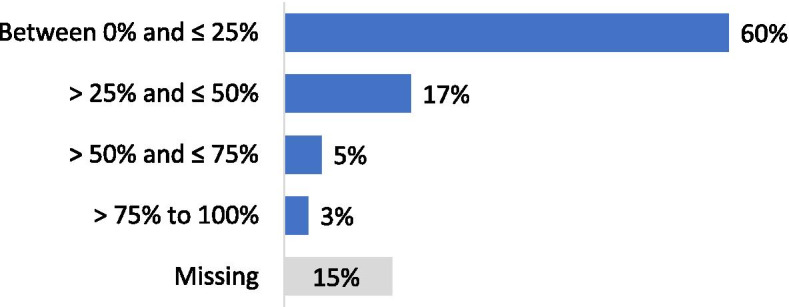
Answers to the question: “How often is meniscus resection performed in conjunction with ACL reconstruction?”

**Fig. 5:**
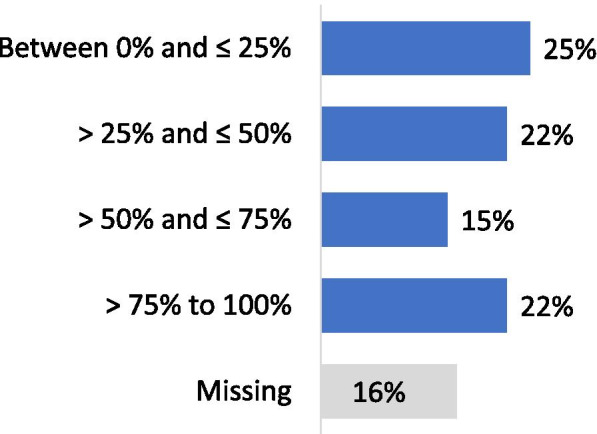
Answers to the question: “How often is meniscus repair performed in conjunction with ACL reconstruction?”

**Fig. 6:**
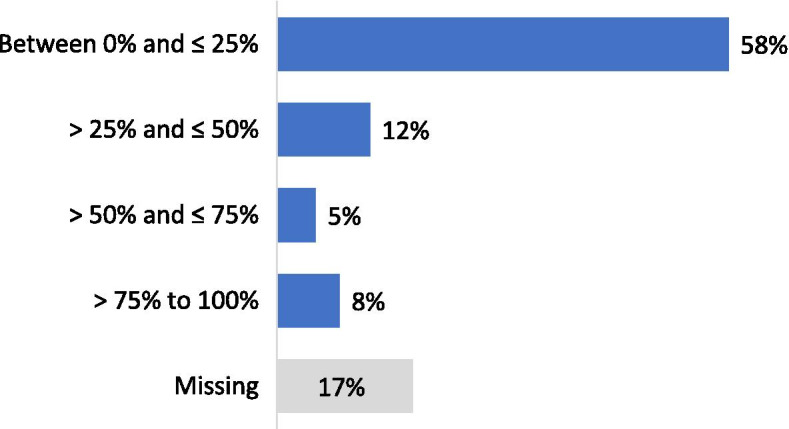
Answers to the question: “What type of meniscus tear do you repair?: Partial resection in degenerative lesions”

Forty-one per cent of the respondents performed less than 100 arthroscopic procedures per year (Fig. 2). For isolated meniscal procedures, 48% of respondents performed less than 50 procedures per year (Fig. 3). Seventy-five per cent of the participants estimated that they were performing more meniscus repair procedures and 85% less APM than 5 years ago. Regarding the management of meniscus tears in association with Anterior Cruciate Ligament Reconstructions (ACLR), 60% of the respondents declared that they perform APM in conjunction with ACLR in less than 25% of cases (Fig. 4). Twenty-five per cent declared to perform meniscus repair in conjunction with ACLR in less than 25% of cases and 37% in more than half of the cases (Fig. 5). Fifty and fifty-one per cent of respondents answered that they would repair respectively a medial or a lateral root tear in less than 25% of cases. Regarding the management of DML’s, 58% of the respondents would perform APM and meniscal repair in less than 25% of cases and only 13% in more than half of the cases (Fig. 6). Finally, 60% and 66% of respondents stated that the ESSKA meniscus consensus on degenerative and traumatic lesions respectively had an impact on their daily clinical practice. Only eighteen and nineteen per cent were not familiar with the consensus.

### National trends

The frequency of both APM and meniscus repair was reported from France, Belgium, Germany and Japan (Table 1).

- a) France [5]

**Table 1:** National trends in meniscus surgery; APM: Arthroscopic Partial Meniscectomy; N/E: Not Evaluated

Countries	Period	APM	Repair
France	2005–2017	- 21.4% (rate)	+ 320% (rate)
Belgium	2007–2017	- 28.6% (rate)	N/E
Germany	2010–2017	- 30% (number)	+ 55% (number)
Japan	2011–2016	91% to 75% (ratio: APM/meniscus procedures)	9% to 25% (ratio: repair/meniscus procedures)

Data were extracted from the French agency for information on hospital care (ATIH) database. The number of procedures was evaluated for APM or meniscus repair between 2005 and 2017. The rate of APM gradually decreased in the entire country from 19.80/10,000 inhabitants in 2005 to 15.77/10,000 inhabitants in 2017, representing a decrease of 21.4%. During the same period, the rate of meniscus repair increased from 0.42/10,000 inhabitants in 2005 to 1.36/10,000 inhabitants in 2017, representing an increase of 320%. Large regional differences were observed: regions in Eastern France had higher APM rates, while regions in Western France had higher meniscus repair rates. When the analysis of procedures between 2008 and 2017 was stratified by age, a similar increase in repair procedures was found in all age categories. Conversely, the reduction of APM was most apparent before the age of 40 years, and the number of APM procedures was stable after 60 years of age.

It’s important to notice that data from ATIH only reflects the encoding process and do not take in account the non-operative treatment of DML’s or meniscus surgeries during ACL reconstruction procedures,

- b) Belgium

Between 2007 and 2017, the rate of APM gradually decreased from 39.1/10,000 inhabitants in 2007 to 27.9/10,000 inhabitants in 2017 (28.6% reduction). Large regional differences were also observed. In 2019, the Flemish region had higher (41.6/10,000 inhabitants) APM rates compared to the region of Wallonia (24.9/10,000 inhabitants). The highest amount of APM concerned patients which were aged between 50 and 59 years. Between 2016–2019, the mean age of patients undergoing APM was 51.6 years. In 2017, meniscus repair procedures were performed in 994 patients comprising 2.5% of all meniscus surgeries. As much as 5% of the national budget in general orthopedic procedures was used for APM.

It’s important to notice that these data only reflect the encoding process, and this process for orthopaedic surgical procedures in Belgium is not attractive for meniscus surgeries (only 7 codes in Belgium vs. 31 for example in Luxembourg).

- c) Germany

Between 2010 to 2017 the number of procedures of isolated meniscus surgery (repairs and APM) were evaluated from the National German Database.

Over this period, the number of APM gradually decreased from approximately 250,000 to 175,000 between 2010 and 2017 (30% decrease) while the number of meniscus repair increased from 13,900 21,400 during the same period (55% increase).

- d) Japan [6]

The number of procedures of isolated meniscus surgery and meniscus repairs were evaluated between 2011 to 2016. Data were extracted from the Japanese National Health Insurance Claims Database and the Statistics of Medical Care Activities Database.

The estimated annual number of meniscus surgeries over the analyzed period ranged from 33,000 to 40,000. The percentage of meniscus repair increased from 9 to 25% between 2011 and 2016. The APM ratio (APM/meniscus procedures) decreased by 16% from 91 to 75% during the same period. The frequency of meniscus surgery showed two peaks, one in adolescent, and the second one in patients in their 60 s. Meniscus repair increased specifically in adolescent from 26 to 53% between 2011 and 2016. Regardless this group of age an increase of meniscus repair was seen the entire population between early 20 s to the late 70 s.

## Discussion

The main finding of the current paper is that there has been a significant shift in the surgical management of meniscal lesions over the last decade with a general trend towards more meniscal repairs and an overall reduction of APM procedures according to ESSKA members as well as to the statistics from 4 specifically examined countries (Belgium, France, Germany and Japan). Eighty percent of respondents were familiar with the ESSKA consensus which established APM not as a first but a second line treatment of DML. This awareness that APM may be of limited value in some patients has led to a general decrease of the annual number of such procedures in the four examined countries. Likewise, more traumatic meniscus tears are being repaired nowadays according to both ESSKA members and national databases, which confirms the hypothesis.

Few studies have looked into the surgical practice for isolated meniscal lesions. An increasing number of isolated meniscal repair procedures (+ 11.4%) was seen between 2005 and 2011 in the USA. At the same time, in the same country, the number of APM procedures increased by 14% showing the abuse of this surgical technique [2]. In England [1], the APM rate increased considerably from 1998 to 2013 as well (going from 5.1/10,000 inhabitants to 14.9) and then decreased slightly until 2017 (to 12/10,000 inhabitants). Unfortunately, this study provided no information on the change in the meniscal repair rate. In Denmark, Thorlund et al. [16] showed that the number of meniscus procedures had doubled between 2000 and 2011 (16.1 to 31.2/10,000 inhabitants), with the largest increase seen in patients > 35 and > 55 years of age. These findings suggest that the increase in procedures mainly impacts patients with degenerative lesions. In another Danish study, Hare et al. [4] reported an increase in the rate of arthroscopic meniscus surgery (repair and APM) in public and private hospitals between 2000 and 2011. The increase was especially noticeable in the private sector, where the proportion of arthroscopic meniscus surgery went from 1 to 32% during this period.

Concerning meniscus procedures in ACL-injured knees during ACL reconstruction, a systematic review of more than 11,000 meniscal tears treated at the time of ACL reconstruction found that for 65%, the meniscus was partially or completely removed, while 26% were treated with repair and 9% were left in situ [11]. In the USA, Mall et al. [10], observed that the number of concomitant APM and ACL reconstruction increased from 36.8% in 1994 to 40.2% in 2006. In the same country, between 2010 and 2018, Partan et al. [12], demonstrated that during the 9-year study period, there was a gradual decrease in the proportion of APM (from 80.8% of concomitant procedures in 2010 to 63.8% in 2018), while the proportion of meniscal repairs almost doubled (from 19.2% in 2010 to 36.2% in 2018) (trend, P < 0.001). In England, Abram et al. [1], observed that the rate of concomitant ACL reconstruction and meniscus repair increased from 0% in 1997/1998 to 10.7% of ACL reconstruction cases in 2016/2017. In a study published by Prentice et al. [14] which investigated 6 registries from different countries in Europe and America, the authors demonstrated that the rate of diagnosed meniscus lesions during ACL reconstruction varies considerably from one country to another (from 37% in United Kingdom to 73% in Luxembourg). This disparity seems to be confirmed by the results of the ESSKA survey where 25% of respondents declared to perform meniscus repair in ACL-associated meniscus injuries in less than 25% of cases and 37% in more than half of the cases. These findings indicate an inhomogeneous surgical approach towards both the identification of meniscus tears and meniscal repair in ACL reconstruction. Indeed, recent studies have established that many meniscus injuries occurring in conjunction with ACL injuries had neither been diagnosed nor treated in the past [9].

Analyzing data from other countries helped to confirm the recent international trends of a decreased number of APM (isolated and during ACL reconstruction procedure) and a greater use of meniscus-preserving repair procedures, especially in younger patients.

## Conclusion

The 2021 ESSKA members' survey as well as statistics from 4 specifically examined countries (Belgium, France, Germany and Japan) suggest there has been a significant shift over the last years in the surgical management of meniscal lesions towards less APM and more conservative treatments. Nevertheless, the findings on meniscus repair identified an inhomogeneous approach for repair of ACL associated meniscus lesions.

## Data Availability

Not applicable.
